# PD-L1 expression and its significance in advanced NSCLC: real-world experience from a tertiary care center

**DOI:** 10.1186/s43046-024-00207-5

**Published:** 2024-01-29

**Authors:** Sindhu Kilaru, Soumya Surath Panda, Lalatendu Moharana, Debahuti Mohapatra, Satya Sundar G. Mohapatra, Adyakinkar Panda, Spoorthy Kolluri, Suma Devaraj, Ghanashyam Biswas

**Affiliations:** 1 Department of Medical Oncology, IMS and SUM Hospital, Siksha ‘O’ Anusandhan University, Bhubaneshwar, Odisha India; 2 Department of Pathology, IMS & SUM Hospital, Siksha ‘O’ Anusandhan University, Bhubaneswar, Odisha India; 3 Department of Radiology, IMS & SUM Hospital, Siksha ‘O’ Anusandhan University, Bhubaneswar, Odisha India; 4 Department of Medical Oncology, Sparsh Hospital, Bhubaneswar, Odisha India

**Keywords:** Lung cancer, Non-small cell lung cancer, PD-L1, Epidermal growth factor receptor, Tobacco use

## Abstract

**Background:**

Targeted therapies against programmed death ligand-1 (PD-L1) in non-small cell lung cancer (NSCLC) have revolutionized the management in recent years. There is paucity of data on the significance of PD-L1 expression in NSCLC from India. We aimed to study the prevalence of PD-L1 expression and its relation with different clinico-pathological parameters in advanced NSCLC from a tertiary care center in Eastern India.

**Methods:**

All consecutive patients with advanced NSCLC diagnosed from January 2020 to December 2021 were prospectively evaluated for PD-L1 expression in formalin fixed-paraffin embedded tumor tissue specimens using immunohistochemistry analysis. A PD-L1 expression of < 1%, 1–49%, and ≥ 50% were considered negative, low, and high expression positive respectively, and association with various parameters was performed.

**Results:**

Out of the 94 patients (mean age 59.6 ± 14 years and 63.8% males), PD-L1 positivity was seen in 42 (44.7%) patients, with low positivity (1–49%) in 29 patients and high positivity (≥ 50%) in 13 patients. Epidermal Growth Factor Receptor (EGFR) mutations were seen in 28 patients (29.8%). There were no significant differences in PD-L1 positivity with respect to gender, age, and molecular mutation status. PD-L1 positivity was significantly associated with tobacco use (*p* = 0.04), advanced tumor stage (*p* < 0.001), and higher nodal stage (*p* < 0.001). Median overall survival in the cohort was 17 months and it was not significantly different between the PD-L1 positive and negative groups.

**Conclusions:**

Forty-five percent of advanced NSCLC patients in our cohort showed positive PD-L1 expression and it is associated with tobacco use and aggressive tumor characteristics.

## Introduction

A growing interest in targeting molecules involved in immune regulation is being generated in the recent times. Programmed death-1 (PD-1) is an immunoinhibitory receptor expressed by activated T-cells and other immune cells. Its engagement by the ligands Programmed death-ligand (PD-L1) or PD-L2 inhibits the kinases involved in T-cell activation and thereby prevents the innate cytotoxic T-cell response against tumor cells [1, 2]. Immunotherapy with anti-PD-L1 or anti-PD-1 antibody overcomes this block on the innate immune system and facilitates it to react with the tumor cells. This new treatment modality of cancer immunotherapy promises to improve the outcomes of patients with advanced cancers. PD-L1 expression and its association with clinico-pathological parameters and outcomes have been a point of interest in studies on several cancers including lung, esophageal, gastric, colorectal, urothelial, and oropharyngeal squamous cell carcinoma. Immunotherapy with PD-1/PD-L1 targeted monoclonal antibodies has changed the therapeutic and prognostic landscape for several cancers. Lung cancer is the leading cause of cancer-related deaths worldwide and as per the Globocan-2020 estimates, lung cancer was the fourth most common cancer in India and second most common cancer among males in the country.

With the results of the CHECKMATE-057 trial [3], Nivolumab received an expanded indication from the United States Food and Drug Administration (FDA) to include all non-small cell lung cancer (NSCLC) after prior platinum-based chemotherapy. With the FDA approval of immunotherapeutic monoclonal antibodies such as nivolumab and pembrolizumab, much attention is generated to study the expression and significance of PD-L1 in lung cancer. Much of the data pertaining to this comes from clinical trials in the West [3–6]. Due to paucity of literature from the Indian subcontinent, we conceptualized this study to assess the expression of PD-L1 in advanced NSCLC and to identify its association with various clinico-pathologic parameters.

## Materials and methods

### Study setting and subjects

All consecutive patients with stage IV adenocarcinoma lung diagnosed and managed at our hospital from January 2020 to December 2021 were screened for enrolment. Those with a prior history of chemotherapy/radiotherapy/alternative treatments and those refusing consent were excluded. After taking informed consent from patients/primary caregivers, relevant history was recorded from the patients as well as the available case records. This included details such as demographic information like age, sex, socio-economic background, clinical manifestations, investigations pertaining to cancer with the information on site, staging, and extent. The details regarding molecular mutation status such as epidermal growth factor receptor (EGFR) mutations, and anaplastic lymphoma kinase (ALK) mutations were also recorded. The follow-up period was calculated from the time of enrolment to the date of last follow-up/death within the time frame of the study duration. Informed consent was obtained from the study participants and the study adhered to the protocols of the Helsinki declaration (modified 2000). The study was approved by the ethics committee of the institute (DMR/IMS.SH/SOA/180471/2021).

### Immunohistochemistry analysis (IHC)

**Fig. 1:**
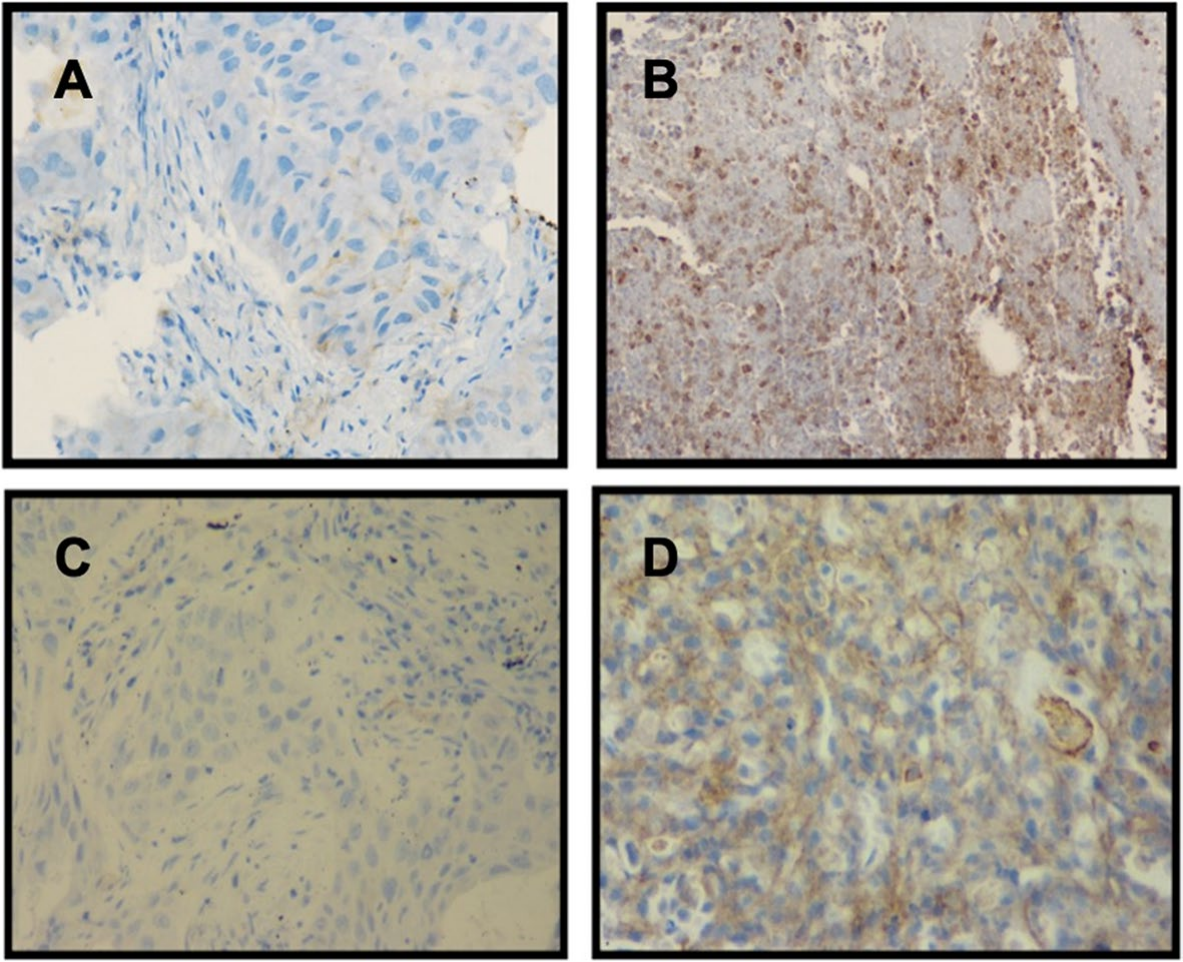
PD-L1 tumor cell score on immunohistochemistry staining of biopsy specimens: **A** 0% by SP263; **B** 35% by SP263; **C** 0% by 22C3; **D** 55% by 22C3

The same tumor tissue sample used for primary histological analysis of the cancer prior to the institution of any kind of treatment was used for the IHC to determine PD-L1 expression. PD-L1 testing was done on the 3 μm tissue sections using rabbit anti-human PD-L1/CD274 monoclonal antibody (SP263, Ventana) on Ventana Benchmark XT Autostainer (Roche, US) and the expression was detected using OptiView DAB IHC Detection kit (Roche, US), and using a mouse monoclonal anti-PD-L1 antibody clone 22C3 pharmDx (Agilent) on Dako Autostainer Link 48 platform (Dako, Inc) as previously described and as per the manufacturer’s instructions [7, 8]. Positive PD-L1 staining is considered complete and /or partial, circumferential or linear plasma membrane staining on tumor cells at any intensity that can be differentiated from background and diffuse cytoplasmic staining. The percentage of tumor cells with positivity was recorded as a tumor cell score. A PD-L1 expression of < 1%, 1–49%, and ≥ 50% was considered negative, low, and high expression positive respectively (representative images in Fig. 1).

### Statistical analysis

The data collected was analyzed using SPSS (Statistical Package for Social Sciences) version 23 (SPSS Inc, Armonk, NY, USA). Descriptive statistics- mean, standard deviation, and proportions were used to summarize the variables if normally distributed, median, and interquartile range were used for skewed data. Frequencies of various findings of tumor characteristics like T stage, N stage, and number of sites of metastasis were calculated as percentages. Comparison of continuous and categorical variables between two groups (PD-L1 positive and negative) was done using Mann Whitney test and chi-square/Fisher exact tests respectively. Survival analysis was done using the Kaplan–Meier method and a comparison of survival outcomes between the groups was done using the log-rank test. All tests were two-tailed and a *p* value of < 0.05 was considered statistically significant.

## Results

A total of 114 patients were diagnosed with stage 4 NSCLC (adenocarcinoma) during the study period. After the exclusion of 10 patients due to nonviable tumor tissue for PD-L1 testing and 10 patients who refused consent for the study, a total of 94 patients were finally analyzed. The mean age of the cohort was 59.6 ± 14 years and there were 60 (63.8%) males. About one-third of the patients were in Eastern Co-operative Oncology Group-Performance Score (ECOG PS) 2 at presentation. The majority of the patients (65%) were assessed using SP-263 (Ventana) IHC assay and 35% underwent PD-L1 estimation using 22C3 (PharmDx, Dako) IHC assay. Table 1 summarizes the baseline characteristics of patients with advanced NSCLC. Of the 94 patients, 28 patients (29.8%) had EGFR mutants; four patients (4.2%) were ALK-positive and one patient (1.1%) was ROS1 positive.

**Table 1:** Characteristics of patients with advanced NSCLC

Age in years (mean ± SD)	59.6 ± 14
Gender (males, *n*, %)	60 (63.8%)
Tobacco use	30 (32%)
Tumor characteristics	
T stage	
a. T2	12 (12.7%)
b. T3	48 (51.1%)
c. T4	34 (36.2%)
N stage	
a. N0	2 (2.1%)
b. N1	8 (8.5%)
c. N2	40 (42.5%)
d. N3	44 (46.8%)
Sites of metastasis	
1. Pleural	41 (43.6%)
2. Brain	11 (11.7%)
3. Skeletal	14 (14.9%)
4. Pleural + brain	5 (5.3%)
5. Pleural + skeletal	18 (19.1%)
6. Brain + skeletal	3 (3.2%)
7. Pleural + brain + skeletal	2 (2.1%)
Stage	
1. IVa	58 (61.7%)
2. IVb	36 (38.3%)
ECOG PS	
a. 0	9 (9.6%)
b. 1	19 (20.2%)
c. 2	31 (33%)
d. 3	25 (26.6%)
e. 4	10 (10.6%)
Molecular mutation status	
1. EGFR	Wild- 66 (70.2%)
	Mutant- 28 (29.8%; *exon 19*–15; *exon 21*–8, *exon 18*–3, *exon 20*–2)
2. ALK	4 (4.2%)
3. ROS1	1 (1.1%)

*NSCLC* non-small cell lung cancer, *ECOG-PS* Eastern Co-operative Oncology Group-Performance Score, *EGFR* epidermal growth factor receptor, *ALK* anaplastic lymphoma kinase, *ROS* Ros proto oncogene-1, receptor tyrosine kinase

Sixty-four patients received first-line chemotherapy with pemetrexed and carboplatin while the remaining patients received kinase inhibitors based on their EGFR/ALK/ROS mutation status (EGFR tyrosine kinase inhibitors-26, Ceritinib-3, Crizotinib-1). PD-L1 positivity was seen in a total of 42 (44.7%) patients, with low positivity (1–49%) in 29 patients and high positivity (≥ 50%) in 13 patients. 50% of males and 35.3% of females showed PD-L1 positivity. Similarly, 37% of those aged < 60 years and 49% of those aged > 60 years showed PD-L1 positivity. There were no statistically significant differences in PD-L1 positivity with respect to sex and age group. We found a significant difference in PD-L1 positivity with respect to tobacco usage with a higher percentage of tobacco users being PD-L1 positive compared to non-users (60% vs 37.5%; *p* = 0.04). Coming to tumor characteristics, patients with tumor stage of ≥ T3 had higher PD-L1 positivity when compared to those with lower T stages (58% vs 8%; *p* < 0.001). Similarly, those with higher nodal stage (N2 and above) had more PD-L1 positivity compared to those with lower nodal stage (54% vs 5.5%; *p* < 0.001). No significant differences in PD-L1 positivity were observed with regards to the site of metastasis and the number of sites of metastases (*p* > 0.05).

**Fig. 2:**
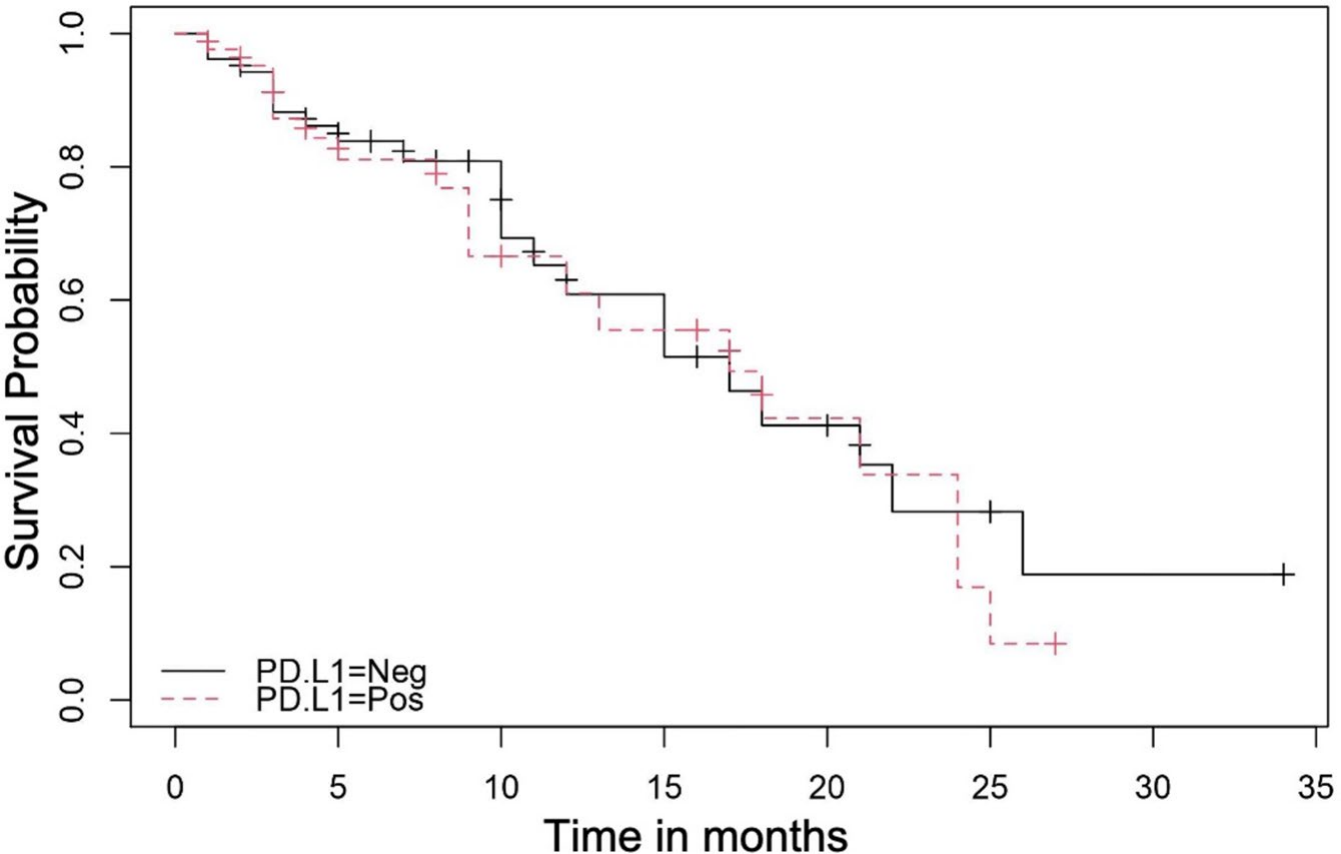
Kaplan–Meier curves showing overall survival in PD-L1 positive and negative groups

On comparing the molecular mutation status and PD-L1 positivity, we found that 46.4% of EGFR-positive and 44% of EGFR-negative patients showed PD-L1 positivity, which was not statistically significant (*p* = 0.8). Out of the four ALK-positive patients, two were PD-L1 positive and the only ROS-positive patient was also PD-L1 positive (≥ 50%). Table 2 summarizes the comparison of various parameters between PD-L1 positive and negative groups. A sub-group analysis of high and low positive PD-L1 patients was done and no significant differences were found in age, sex, molecular mutation profile, and tumor characteristics between these sub-groups. Median overall survival (OS) in the advanced NSCLC cohort was 17 months (range 1–34 months), irrespective of the treatment received. On comparison of OS between PD-L1 positive and negative groups, no significant difference was observed (log-rank *p* = 0.6; Fig. 2).

**Table 2:** Comparison of characteristics between PD-L1 positive and negative groups

	**PD-L1 negative (*n* = 52)**	**PD-L1 positive (*n* = 42)**	***p* value**
Age > 60 years	50.8% (30)	49.2% (29)	0.25
Males	50% (30)	50% (30)	0.16
Tobacco users	40% (12)	60% (18)	**0.04**
T stage			** < 0.001**
1. T1 + T2	23 (44.2%)	2 (4.7%)	
2. T3 + T4	29 (55.8%)	40 (95.2%)	
N stage			** < 0.001**
1. N0 + N1	17 (32.7%)	1 (2.4%)	
2. ≥ N2	35 (67.3%)	41 (97.6%)	
Stage			0.21
1. IVa	35 (67.3%)	23 (54.7%)	
2. IVb	17 (32.7%)	19 (45.2%)	
Metastasis sites > 2	15 (53.6%)	13 (46.4%)	0.82
EGFR mutation positive	15 (53.6%)	13 (46.4%)	0.82

## Discussion

Much of the data on the prevalence of PD-L1 expression in patients with NSCLC comes from clinical trials of selected patients and ranges from 25 to 60% based on the IHC cut-offs used for defining positivity. In the CHECKMATE-057 trial, which included patients with advanced non-squamous NSCLC, the prevalence of PD-L1 positivity was 36% when a cut-off of ≥ 10% was used, but it increased to 40% when the cut-off was reduced to ≥ 5% [3]. If the cut-off ≥ 1% is used, the prevalence greatly increases to more than 60% [4, 5]. In clinical studies by Gettinger et al. [9, 10] and Yang et al. [11], the prevalence of PD-L1 positivity ranged from 25 to 59% using ≥ 5% as a cut-off. Nevertheless, a cut-off of ≥ 1% is now used as a standard by many researchers to determine positivity on IHC. In a large-scale real-world study involving 791 patients of NSCLC, a PD-L1 positivity of 63% was identified [12]. The prevalence also differs due to the different antibody clones used for IHC. We used two different IHC clones- SP263 and 22C3 in our study and this was unlikely to affect our results as the concordance rates of results between the different IHC platforms was assessed and proved to be very good. The Blueprint study has been undertaken to investigate the potential interchangeability and agreement among these different IHC assays. The results from the Blueprint phase-1 study demonstrated that three PD-L1 assays (22C3, 28–8 and SP263) showed comparable analytical performance for assessment of PD-L1 expression on tumor cells, whereas the SP-142 PD-L1 assay appeared to stain fewer tumor cells compared with the other assays [13]. These results were further validated by the Blueprint phase-2 study which showed highly comparable staining by the 22C3, 28–8 and SP263 assays [14]. The International Association for the Study of Lung Cancer (IASLC) pathology committee has set up some guidelines on reporting the PD-L1 IHC assays, irrespective of the clone used. It recommends that PD-L1 expression should be reported according to the interpretation manual or guide for each assay.

Our cohort of stage IV NSCLC showed a higher percentage of PD-L1 positivity when compared to similar real-world studies involving stage IV NSCLC patients by Yan Jin et al. [15] and Pawelczyk et al. [16]. From the available scarce literature on Indian studies, the prevalence of PD-L1 positivity ranges from 27 to 53% [17–20]. Our results are similar to these studies, with PD-L1 positivity being 45% in our cohort of patients with advanced NSCLC. Many researchers tried to identify the relation between PD-L1 positivity and various clinic-pathological parameters like age, sex, tumor characteristics, and grade; however, the results from these studies are contrasting. While some studies showed higher male preponderance in PD-L1 positivity [16, 21–23], other studies showed no such association with gender [12, 24–26]. We did not identify any association with age and sex in our study.

We observed that tobacco users had a higher prevalence of PD-L1 positivity compared to non-users which was statistically significant (60% vs 37.5%; *p* = 0.04). This corroborates with results from previous studies [15, 21, 23, 27]. In a recent meta-analysis involving 1981 patients of NSCLC, the response to treatment with PD-1 inhibitors was more efficacious in NSCLC patients with smoking history compared to non-smokers [28]. This has been linked to the alteration of anti-tumor response through regulatory T-cells and natural killer cells in smokers. Another interesting observation in our study was the significant association of PD-L1 positivity with higher tumor and nodal stage. Patients with higher tumor stage (≥ T3) had higher PD-L1 positivity when compared to those with lower T stages (58% vs 8%; *p* < 0.001). Similarly, those with higher nodal stage (≥ N2) had more PD-L1 positivity compared to those with lower nodal stage (54% vs 5.5%; *p* < 0.001). In a large study from South Korea involving 785 lung adenocarcinoma patients, the authors found a significant association of PD-L1 positivity with higher T, N, and M stages [27]. Another large study from China [21], with 847 adenocarcinoma lung patients, identified that significantly more T3/T4 than T1/T2, and more patients with lymph node metastasis showed positive PD-L1 staining (p < 0.001). In contrast to the results from Indian studies [18, 20], we also had similar results as reported in the above-mentioned studies besides a few more [15, 16, 23, 29].

It was observed in recent times that PD-L1 expression is upregulated by EGFR mutations in NSCLC [30, 31]. Nan Chen et al. [32] described the molecular pathway of this upregulation in their study and found that PD-L1 expression can be induced by EGF stimulation, and exon-19 deletions, among others. This PD-L1 expression mediated by EGFR activation induces apoptosis of T-cells through the PD-L1/PD-1 axis in tumor cells. In one recent study, PD-L1 positive patients treated with EGFR-targeted drugs had higher response rates and longer time to progression compared with PD-L1 negative patients [33]. While few studies documented an association between PD-L1 expression and molecular alterations such as EGFR mutations, ALK, and KRAS [33–35], other studies have failed to find such associations [25, 26, 36, 37]. Our study did not demonstrate a significant association of PD-L1 positivity with EGFR, ALK, and ROS mutations. PD-L1 expression is also being used as a prognostic marker and its role in still unclear. Few studies identified its association with improved OS [6, 38, 39] while others found either no association or worse OS and outcomes [29, 31, 40]. We did not find any difference in OS between PD-L1-positive and PD-L1-negative patients. Also, there was no significant difference in OS between low- and high-positive PD-L1 patients.

The main limitation of our study is that only a handful of patients received immunotherapy after the PD-L1 testing, owing to its high costs and financial constraints present for the majority of our patient cohort. We could not study the role of immunotherapy and its association with various factors. In spite of the limitations, ours is a large prospective study on PD-L1 expression in advanced NSCLC from Eastern India.

## Conclusion

To conclude, nearly half of advanced NSCLC patients have evidence of PD-L1 expression and it is associated with tobacco use and aggressive tumor characteristics. The findings of our study will significantly add to the very limited literature available on the role of PD-L1 testing in these solid cancers from the Indian sub-continent and we opine that further large-scale prospective real-world studies are needed to clearly elucidate the role of PD-L1 as a prognostic marker in advanced NSCLC.

## Data Availability

Data cannot be shared openly to protect study participant privacy as per our institutional policies, however is available from the corresponding author on reasonable request.

## Abbreviations

PD-L1: Programmed death-ligand 1
NSCLC: Non-small cell lung cancer
EGFR: Epidermal growth factor receptor
PD-1: Programmed death-1
ALK: Anaplastic lymphoma kinase
IHC: Immunohistochemistry
ECOG-PS: Eastern Co-operative Oncology Group-Performance Score
IASLC: International Association for the Study of Lung Cancer (IASLC)
OS: Overall survival
